# Radiotherapy of High-Grade Gliomas: First Half of 2021 Update with Special Reference to Radiosensitization Studies

**DOI:** 10.3390/ijms22168942

**Published:** 2021-08-19

**Authors:** Guido Frosina

**Affiliations:** Mutagenesis & Cancer Prevention Unit, IRCCS Ospedale Policlinico San Martino, Largo Rosanna Benzi 10, 16132 Genova, Italy; guido.frosina@hsanmartino.it; Tel.: +39-010-555-8543

**Keywords:** high-grade-glioma, radiotherapy, update, sensitization

## Abstract

Albeit the effort to develop targeted therapies for patients with high-grade gliomas (WHO grades III and IV) is evidenced by hundreds of current clinical trials, radiation remains one of the few effective therapeutic options for them. This review article analyzes the updates on the topic “radiotherapy of high-grade gliomas” during the period 1 January 2021–30 June 2021. The high number of articles retrieved in PubMed using the search terms (“gliom* and radio*”) and manually selected for relevance indicates the feverish research currently ongoing on the subject. During the last semester, significant advances were provided in both the preclinical and clinical settings concerning the diagnosis and prognosis of high-grade gliomas, their radioresistance, and the inevitable side effects of their treatment with radiation. The novel information concerning tumor radiosensitization was of special interest in terms of therapeutic perspective and was discussed in detail.

## 1. Introduction

The earliest surgical interventions for high-grade glioma (HGG) date to 1884 and resulted in the patient’s death less than a month after surgery [1]. During the following 137 years, advancements in surgical technique, imaging, anesthesia, control of cerebral edema, introduction of magnetic resonance imaging (MRI), and adjuvant therapies allowed improving outcomes, but the median survival for patients affected by these grades III and IV tumors (42 and 14 months respectively) remain discouragingly brief [2]. The HGG highly infiltrative and complex microenvironment phenotypes, multiple resistance mechanisms, and the anatomical obstacle represented by the blood–brain barrier represent formidable challenges to develop effective treatments [3]: as a consequence, therapeutic guidelines have been essentially unchanged, since the introduction of temozolomide by Roger Stupp et al. more than fifteen years ago [4]. The current standard treatment involves maximally safe surgical resection followed by radiotherapy (RT) over a 6-week period with concomitant temozolomide (TMZ) chemotherapy followed by TMZ maintenance [5]. This therapy is seldom definitely effective, being almost invariably followed by tumor recurrence and progression. Several HGG patients are not suited for guidelines therapy and can only be subjected to palliation.

According to the Oxford Centre for Evidence-based Medicine, there is level 1 evidence for the use of RT in previously unirradiated HGG patients, with numerous randomized controlled trials showing a clear survival benefit (ref. [6,7] for relevant historical perspectives). The majority of the trials had combined grade 3 and 4 gliomas, although the majority of the patients had grade 4 disease [7]. The current guidelines for those HGG patients to be treated for the first time with RT involve a total 60 Gy split in 2.0 Gy fractions delivered on weekdays for six weeks. Thereafter, almost invariably, the tumor relapses and progresses and how to treat the recurrent patients is far from being established. Although RT techniques have steadily evolved with time and several of them are now explored for HGG recurrent patients including hypofractionated RT, tumor-treating fields (TTFs), stereotactic radiosurgery (SRS), and others, little or no survival benefit has been so far achieved [6,7,8]. Hence, there are still many questions to be answered about the efficacy and toxicity associated with a second course of radiation. Despite their low incidence rate, HGG remain a major health issue [9,10]. The first semester 2021 research advances on HGG, with special reference to radiotherapy (RT), are discussed in the following.

## 2. Diagnosis and Prognosis

Unlike primary HGG that usually develop ex novo in the elderly, secondary HGG may often progress from lower-grade gliomas [11]. In addition to those variations of tumor natural history, radiation necrosis and pseudo progression in MRI may also complicate diagnosis [12]. To limit those drawbacks, since 2016, the classification of tumors of the central nervous system (CNS) by the World Health Organization (WHO) includes some molecular parameters in addition to the traditional microscopic characteristics [13]. Thus, molecular markers have added a level of objectivity that was previously lacking for some categories of CNS tumors [13,14]. Similarly, the joint guideline committee of the Chinese Glioma Cooperative Group, the Society for Neuro-Oncology of China, and the Chinese Brain Cancer Association updated their clinical practice guidelines [15] providing recommendations for the diagnostic and management decisions as well as limiting unnecessary treatments and costs [15]. It was concluded that WHO grade II/III isocitrate dehydrogenase (IDH)-wildtype diffuse astrocytoma containing telomerase reverse transcriptase (TERT) promoter mutations, chromosome 7 gain/10 loss, and/or EGFR amplification correspond to a WHO grade IV diagnosis and should be classified as diffuse astrocytic glioma, IDH-wildtype, with molecular features of glioblastoma [GB-WHO grade IV (DAG-G)] [16]. Compared to patients with classic IDH-mutant astrocytoma, the mean age is older, tumors are more diffuse, and overall survival (OS) is significantly shorter [16]. Importantly, IDH genotypes and other molecular characteristics can now be identified by radiomics features from multiparameter MRI, thus further increasing pre-and post-operative diagnostic possibilities [17,18,19]. Merely to quote one couple of examples, most non-astrocytic tumors use TERT expression for telomere maintenance-mediated resistance, while HGG use the alternative lengthening of telomeres (ALT) pathway [20]. TERT and ALT are associated with unique magnetic resonance spectroscopy-detectable metabolic signatures in genetically engineered and patient-derived glioma models. Hyperpolarized [1-(13)C]-alanine flux to pyruvate was proposed as an imaging biomarker of ALT status and hyperpolarized [1-(13)C]-alanine flux to lactate was proposed as an imaging biomarker of TERT status in brain tumors [20].

The feasibility of using hyperpolarized [1-(13)C] pyruvate for differentiating radiation necrosis from brain tumors has been investigated in vivo by Park et al. [21]. Conventional MRI exhibited typical radiographic features of radiation necrosis and brain tumor with large areas of contrast enhancement and T2 hyperintensity in all animals. Lactate and pyruvate in radiation necrosis were significantly lower than those in glioma and metastatic tissue. These results were consistent with histological findings where tumor-bearing brains were highly cellular, while irradiated brains exhibited reparative changes from radiation necrosis. Hyperpolarized (13)C MR metabolic imaging of pyruvate was proposed as a noninvasive imaging method to differentiate between radiation necrosis and brain tumors [21].

Elderly patients (defined as age ≥ 65), patients with poor performance status (defined as Karnofsky performance status (KPS) < 60 or Eastern Cooperative Oncology Group Performance Status (ECOG PS) > 2), regardless of age and those where brainstem infiltration occurs, all have worse outcomes [22]. Additional negative prognostic markers include p53 mutations, CXCL10 expression, and possibly the male gender [23,24,25].

The mutational spectra following RT in 190 paired primary and recurrent gliomas and 3693 post-treatment metastatic tumors were analyzed by Kocakavuk et al. [26]. RT was associated with significant increases of small deletions (5–15 bp) and large deletions (20+ bp to chromosome-arm length). Small deletions were characterized by a larger span size and were genomically more dispersed when compared to pre-existing deletions and deletions in non-irradiated tumors. Mutational signature analysis implicated classical non-homologous end-joining-mediated DNA repair. A high IR-associated deletion burden was associated with worse clinical outcomes, suggesting that the effective repair of IR-induced DNA damage is detrimental to patient survival [26].

A mechanism-based, mathematical model that characterizes glioma cells’ temporal response to single-dose RT in vitro by explicitly incorporating time-dependent biological interactions with IR was developed by Liu et al. [27]. The proposed model could effectively predict the temporal development of 9L and C6 glioma cells in response to a range of single-fraction IR doses, providing an experimental mathematical framework that allows for quantitative investigation of cells’ temporal response to IR [27].

The neurovascular units responsible for functional connectivity may persist in the HGG tumor albeit with altered magnitude [28]. It has been hypothesized that the strength of functional connectivity within HGG may be predictive of OS. Functional connectivity regions of interest were predefined in de novo HGG patients to characterize the presence of within-tumor functional connectivity observable via functional MRI and investigated in relationship with survival outcomes. Fifty-seven HGG patients were analyzed. Functional connections could be routinely found within the tumor mass and did not significantly correlate to tumor size. Higher functional connectivity strength within HGG tumors was associated with better OS, indicating that functionally intact regions may persist within HGGs and the extent to which functional connectivity is maintained may carry prognostic value [28].

## 3. Advances in RT

### 3.1. Radioresistance

#### 3.1.1. Preclinical Studies

HGGs are made up of multiple populations of tumor cells, among which those with the ability to initiate tumor development, herein referred to as glioma initiating cells (GIC), are of utmost importance. GICs often exhibit stem cell properties and may be constitutively less sensitive to radiation than the tumor mass, thus leading the tumor relapse after the end of the radiotherapy cycle. It is unclear by which mechanisms GICs resist the toxic effects of radiation, although DNA repair is probably one of the mechanisms involved [29]. To identify the factors that determine the radioresistance of HGGs, human and murine radioresistant GICs were generated by exposing them to repeated irradiation cycles [30]. The acquired radio resistance was accompanied by a reduction in proliferative capacity as well as an increase in intercellular adhesion and N-cadherin expression. Conversely, radioresistant GICs lost their acquired phenotype, following CRISPR/Cas9 knockout of N-cadherin. In turn, N-cadherin overexpression was stimulated by the radiation-induced secretion of insulin-like growth factor 1 [30].

PARK7 is a ubiquitous human protein involved in many cellular processes including cell proliferation, transcriptional regulation, cell differentiation, protection from oxidative stress, and maintenance of mitochondrial function. Its possible effects on the stemness and radioresistance of GICs has been studied by Kim et al. [31]. PARK7 was found to be expressed more in GICs than in differentiated cells. Immunohistochemical techniques showed a greater expression of PARK7 in HGG tissues than in normal brain tissues. The shRNA-mediated knockdown of PARK7 inhibited self-renewal of GICs in vitro. Furthermore, the knockdown of PARK7 suppressed the invasive capacity of GICs and increased the sensitivity of GICs to IR. PARK7 suppresses the expression of some stem cell markers including nestin, the epidermal growth factor receptor variant III (EGFRvIII), SOX2, NOTCH1, and OCT4. Conversely, the overexpression of PARK7 in non-GIC CD133 (-) increased self-renewal activities, migration, and IR resistance. Most important of all, PARK7 knockdown increased mouse survival and IR sensitivity in vivo [31].

HGG tumor recurrence occurs mainly in the peritumoral brain zone [32]. The mRNA expression of selected genes including SERPINA3 from microarray data were validated in the tumor core and peritumoral brain zone. Albeit its protein product alpha-1-antichymotrypsin has unclear functions, the SERPINA3 gene knockdown in vitro caused decreased HGG tumor cell proliferation, invasion, migration, transition to mesenchymal phenotype, stemness, and radioresistance. SERPINA3 protein expression was higher in peritumoral brain zone compared to tumor core and also was higher in older patients and IDH wild type and recurrent tumors. Its expression showed positive correlation with poor patient prognosis [32].

Both the perivascular niche and the integration into multicellular networks by tumor microtubes extension may be associated with resistance to therapies in HGG [33]. By long-term tracking of tumor cell fate and dynamics in the live mouse brain, differential therapeutic responses in both niches could be studied. Both the perivascular niche, a preferential location of long-term quiescent GICs, and network integration facilitate resistance against the cytotoxic effects of RT and chemotherapy. Perivascular HGG cells are particularly able to actively repair IR-induced damage. The population of the perivascular niche and resistance in it depend on proficient NOTCH1 expression, whilst NOTCH1 downregulation induces resistant multicellular networks by tumor microtubes extension [33].

Prohibitin regulates mitochondrial ROS production in GICs, thus facilitating their RT resistance [34]. Prohibitin may be found upregulated in GICs and is associated with malignant gliomas progression. Prohibitin binds to peroxiredoxin3, a mitochondrion-specific peroxidase, and stabilizes peroxiredoxin3 protein through the ubiquitin–proteasome pathway. Knockout of prohibitin dramatically elevates ROS levels, thereby inhibiting GIC self-renewal. The deletion or pharmacological inhibition of prohibitin slows tumor growth and sensitizes tumors to RT, thus providing significant survival benefits in GIC-derived orthotopic tumors and GB patient-derived xenografts models [34].

The interaction between the SP1 protein and the pyrine domain of the NLR family (NLRP6) and their role in glioma cell activity has been studied by Yu et al. [35]. SP1 and NLRP6 were abundantly expressed in HGG cells and correlated with patients’ poor prognosis. Under-expression of SP1 reduced the proliferation, migration, and invasiveness of U87 glioma cells in vitro as well as their tumorigenesis in vivo. Cell malignancy was restored after NLRP6 upregulation. Under-expression of SP1 in glioma cells also led to increased proliferation of CD8 (+) T cells and reduced the radio-resistance of U87 cells. Although U87 cells may represent an unreliable HGG model [36], this study suggested that SP1 may interact with the NLRP6 inflammasome to increase the malignancy, immune evasion, and radioresistance of glioma cells [35].

Irradiation of the brain can lead to the onset of a tumor-friendly microenvironment. Astrocytes, when pre-irradiated, increase the stemness and survival of co-cultured glioma cells [37], and mice subjected to IR prior to glioma cell implantation may develop more aggressive tumors. The extracellular matrix of irradiated astrocytes, and in particular its transglutaminase 2 component, were found to be important factors in promoting the stemness and aggressiveness of HGGs. Transglutaminase 2 levels are increased after IR in vivo and in recurrent HGG, and transglutaminase 2 inhibitors can counteract glioma proliferation [37]. The impact of RT on the microenvironment of experimental HGGs may also involve immune suppression [38]. Mice harboring neurosphere/CT-2A HGGs received RT (4 Gy, single dose), as monotherapy. RT reduced tumor-associated macrophages/microglia and monocytic myeloid-derived suppressor cells compared to controls [38].

Hypoxia (O_2_ partial pressure lower than 10 mmHg) is a possible radioresistance factor in HGG [39]. A NIR fluorescent hypoxia-sensitive smart probe (NO_2_-Rosol) for identifying hypoxia was developed via selectively imaging nitroreductase activity, which could correlate to oxygen deprivation levels in cells [40]. This technique was validated in vivo using GBM38 animal models [40].

#### 3.1.2. Clinical Studies

As aforementioned (1. Introduction), several studies have suggested the feasibility of reirradiation of patients with recurrent HGG [41]; however, the actual benefits associated with such repeated courses of IR are questioned by multiple radioresistance mechanisms arising during the first RT course [8]. Thus, for recurrent HGG patients, no guidelines are available for IR treatment, which may presently include conventional RT, three-dimensional conformal RT, intensity-modulated RT, stereotactic radiosurgery, stereotactic RT, and tumor-treating fields [8,39,42]. Survival outcomes and prognostic factors of salvage re-irradiation in recurrent/progressive HGG patients have been investigated by Gupta et al. [43]. The first course of RT had been delivered to a median dose of 59.4 Gy. The median time to recurrence/progression was 4.3 years, while the median time to re-irradiation was 4.8 years. Re-irradiation was delivered with intensity-modulated RT using 1.8 Gy/fraction to a median dose of 54 Gy. At a median follow-up of 14 months after re-irradiation, the 1-year Kaplan–Meier estimate of post-re-irradiation OS was 61.8% [43].

The efficacy and safety of hypo fractionated stereotactic radiosurgery for the recurrence of HGG has been investigated by Guan et al. [44]. A total of 70 patients were included in their study. Forty-nine had an initial diagnosis of GB, and the rest (21) were confirmed to be WHO grade III gliomas. The median prescribed dose was 24 Gy in four fractions. The median OS after treatment was 17.6 months (19.5 and 14.6 months for grade III and IV gliomas, respectively). No elevated toxicities were recorded. Multivariate analysis showed that concurrent bevacizumab with radiosurgery and KPS  >  70 were favorable prognostic factors. A prospective phase II study (NCT04197492) is ongoing to further investigate the value of hypo fractionated stereotactic radiosurgery in recurrent HGG [44].

The efficacy of gamma knife radiosurgery as a salvage therapy for HGG has been investigated by Zhao et al. [45]. The median OS after gamma knife radiosurgery was 13 months. The 1-, 2-, and 5-year survival rates were 51.4, 10.0, and 2.9%, respectively. As expected, the tumor grade and KPS were prognostic factors [45].

A phase III clinical trial has shown that tumor treating fields (TTFs) monotherapy provides comparable survival benefits to chemotherapy in recurrent HGG [46]. However, patients did not equally benefit from TTFs, highlighting the importance of identifying predictive biomarkers of TTFs efficacy. In a retrospective review of an institutional database, 149 recurrent HGG patients were identified of which 29 (19%) were treated with TTFs. Among them, post-progression survival was improved in PTEN-mutant versus PTEN-WT recurrent HGG [46].

Advancements in definition of the RT planned target volume (PTV) have been obtained by different MRI techniques such as ultrahigh-field 7-T MRI, diffusion MRI, perfusion-weighted imaging, and magnetic resonance spectroscopy, which may also aid for predicting tumor grades and molecular subtypes [47]. PET imaging may also importantly contribute to RT treatment planning [48]. PET has been used after RT for response assessment and to distinguish tumor progression from pseudoprogression or radiation necrosis [49]. The Response Assessment in Neuro-Oncology working group has provided a summary of the literature and recommendations for the use of PET imaging for RT of patients with HGG based on published studies, constituting level 1–3 evidence [48,49].

The prompt delivery of RT treatment after surgery is essential to combat tumor radioresistance. A retrospective study to evaluate if time to chemoradiation after surgery is correlated with progression-free survival (PFS) of HGG patients has been conducted in Argentina [50]. Median time to chemoradiation after surgery was 8 weeks for the global population. There was a statistically significant effect of time to chemoradiation on patient PFS. Patients had a PFS of 10 months when they received chemoradiation <5 weeks vs. a PFS of 7 months when they received chemoradiation between 5 to 8 weeks and a PFS of 4 months when they received chemoradiation >8 weeks after surgery [50]. Toor et al. in the U.S. found that RT was started at mean 46 and 85 days after surgery in a managed health care organization and county hospital respectively, and that this time was inversely correlated to time to death that was 412 and 343 days, respectively [51].

Yan et al. first proposed the concept of adaptive RT [52]. Adaptive RT consists of a “variable set-up” RT with improved precision in which computed tomography (CT)/MR images and other types of images are acquired at successive times; anatomical, physiological, and other conditions (e.g., changes in tumor size, morphology, and location) are observed and evaluated; the differences between the original RT plan and the potentially subsequent one are analyzed; the most appropriate doses to be administered after the change in the volume, position, and morphology of the tumor and of normal tissues and organs are determined. This can not only increase the dose that can be administered to the tumor but also minimize the dose radiated to surrounding normal tissues, thus reducing the incidence of complications and improving the long-term quality of life of patients. The appropriate timing of adaptive RT for HGG has been investigated by Cao et al. [53]. Ten patients with HGG were screened and underwent CT/MRI scans before RT and after 10, 20, and 30 fractions of RT. Then, the differences in dosimetry in the target volume and in the organs at risk were compared, and the corresponding RT plans were updated. Adaptive RT could progressively improve the efficacy of irradiation of the target volume of HGGs and in parallel reduce the irradiation dose to organs at risk [53]. Tsien et al. have reported that intensity-modulated RT with an escalated dose may improve the survival of GB patients [54]. The PTV1 (gross tumor volume (GTV1 + 1.5 cm)) received 60 Gy/30 fractions, and the PTV2 (residual tumor or surgical cavity) received 66–81 Gy using simultaneous integrated boosts with concurrent and adjuvant TMZ chemotherapy. The median OS and PFS durations were 20.1 and 9.0 months, respectively, which was superior to the results of Stupp’s protocol [55]. Whether adaptive RT can improve the local control of HGG and increase OS and PFS of patients certainly requires further studies.

Bevacizumab was approved for recurrent HGG on the basis of two phase 2 trials that evaluated its efficacy in patients with recurrent GB [56]. In 2014, two phase 3 trials revealed improved PFS, but not OS, after bevacizumab therapy combined to RT-TMZ [57,58]. Recent analyses on limited groups of patients confirmed the efficacy of bevacizumab in improving the PFS of recurrent HGG patients with an acceptable toxicity profile [56,59]. Currently, the combination of re-irradiation with bevacizumab for recurrent HGG is common, but its efficacy is highly variable. The subject was analyzed in a systematic review by Kulinich et al. [60]. Data on 1399 patients were analyzed, with 954 patients receiving re-irradiation alone and 445 patients receiving re-irradiation + bevacizumab. All patients had initially undergone standard-of-care therapy for their primary HGG. In contrast to previous studies, a multivariate analysis adjusted for median patient age, WHO grade, RT dosing, re-RT fractionation regimen, time between primary and re-irradiation, and re-irradiation target volume showed that bevacizumab therapy was associated with significantly improved OS (2.51, 95% CI (0.11, 4.92) months, *p* = 0.041) but no significant improvement in PFS (1.40, 95% CI (−0.36, 3.18) months, *p* = 0.099). Patients receiving bevacizumab also had significantly lower rates of radionecrosis (2.2% vs. 6.5%, *p* < 0.001) [60]. Thus, the effectiveness of combining bevacizumab to RT in HGG therapy remains an open question [61], and further randomized, genetically characterized studies will be required to clarify what patients may actually benefit from bevacizumab treatment.

In the pediatric setting, brain cancers represent the most frequent solid tumors and a leading cause of cancer-driven mortality. Among pediatric HGG, diffuse intrinsic pontine gliomas (DIPGs) have very poor prognosis, being localized to the brain stem where surgery is often impossible due to the tumor critical location and highly infiltrating nature. Outcomes in children and adolescents with recurrent or progressive HGG may be therefore even poorer than in adults [10,62] and innovative approaches such as e.g., tumor conversion to an immunologically “hot” phenotype have been performed by oncolytic virotherapy [63].

A recent meta-analysis examined the effectiveness of RT in conjunction with TMZ in improving the prognosis of DIPG. A total of 14 studies were considered involving 283 cases of patients with DIPG who were treated with RT in conjunction with TMZ. The pooled 1- and 2-year OS of this treatment were 43% and 11%, respectively [64]. Twenty pediatric patients of recurrent DIPG with a median age of 7.5 years treated with re-irradiation were reviewed retrospectively. Survival endpoints included cumulative OS and re-irradiation OS (re-irradiation starting to death). Seventeen of them (85%) were considered clinical responders with re-irradiation OS ranging 5.3–7 months, depending on the dose [65].

In a cohort of 97 pediatric patients identified with a histologically confirmed primary high-grade spinal glioma, univariate, multivariate, and Kaplan–Meier log-rank testing failed to demonstrate an association between performing surgery, extent of resection, RT, or chemotherapy with improved survival outcomes [66]. Therefore, the risks and side effects of these treatment modalities must be carefully considered in these patients [66].

### 3.2. Radiosensitization

#### 3.2.1. Preclinical Studies

**Figure 1:**
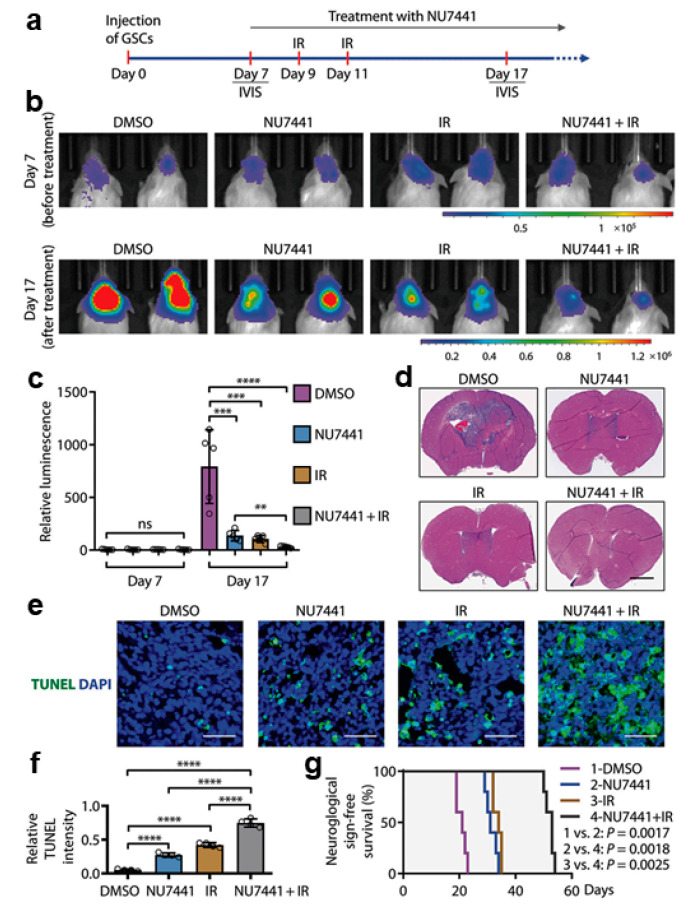
Inhibiting DNA-PKcs in combination with IR improved GB treatment. (**a**–**c**) In vivo bioluminescent imaging of orthotopic GB xenografts derived from H2S GICs expressing luciferase in immunocompromised NSG mice. Seven days after GIC transplantation, NSG mice were treated with DNA-PKcs inhibitor (NU7441) or DMSO (control) by intraperitoneal injection and/or irradiated (IR; 2 × 2 Gy) on day 9 after GIC transplantation. Representative images (**b**) of the four indicated treatment groups on day 7 (before treatment) and day 17 (after treatment) and quantifications (**c**) of tumor growth in mouse brains are shown. Data are means ± SD. n = 5 mice per group. ANOVA analysis was used to assess the significance. **, *p* < 0.01, ***, *p* < 0.001, and ****, *p* < 0.0001. (**d**) Representative images showing H&E staining of cross-sections of mouse brains bearing GIC-derived tumors from four groups of mice treated with NU7441, IR (irradiation), NU7441 + IR, or DMSO (control). Scale bar, 2 mm. (**e**,**f**) TUNEL assay detecting apoptosis (green) in the GB xenografts treated with the DNA-PKcs inhibitor NU7441, irradiation (IR), NU7441 + IR, or DMSO (control) and counterstained with DAPI (blue) to indicate nuclei (**e**). Quantifications of cell apoptosis from four treatment groups (**f**). Scale bars, 50 μm. ANOVA analysis was used to assess the significance. Data are means ± SD. n = 4 independent experiments (about 200 cells per arm). ****, *p* < 0.0001. (**g**) Kaplan–Meier survival curves of mice intracranially transplanted with H2S GSCs and treated with the DNA-PKcs inhibitor NU7441, irradiation (IR), NU7441 + IR, or DMSO (control). n = 5 animals per group. Log-rank analysis was used. Control versus NU7441, *p* = 0.0017; control versus IR, *p* = 0.0017; control versus NU7441 + IR, *p* = 0.0017; NU7441 versus NU7441 + IR, *p* = 0.0018; and IR versus NU7441 + IR, *p* = 0.0025 (modified after [67] with permission).

SOX2 is an important transcription factor for maintaining the stemness of GICs. Fang et al. demonstrated that the DNA-dependent protein kinase (DNA-PK) regulates the stability of SOX2 through its phosphorylation, resulting in the maintenance of GICs [67]. In this regard, phosphorylation of the S251 of SOX2 plays a particularly important role. It was investigated whether the pharmacological inhibition of DNA-PKcs could reduce the radioresistance of GICs and consequently sensitize experimental GBs to RT (Figure 1). The combined treatment with the DNA-PKcs inhibitor NU7441 and irradiation (2 Gy) more effectively inhibited the growth of GIC-derived GB xenografts in comparison to the single treatments with DNA-PKcs inhibitor or irradiation alone (Figure 1a–c). Histological analyses on brain sections showed that mice treated with the inhibitor and irradiation developed much smaller GB tumors than the individual inhibitor-only or irradiation-only treatment groups (Figure 1d). Furthermore, immunofluorescent analyzes of tumor sections with a TUNEL assay showed that apoptosis was significantly increased in tumors treated with the combined treatment as compared to the single treatments (Figure 1e,f). Consistently, the group of mice treated with the DNA-PKcs inhibitor NU7441 and irradiation survived longer than those treated with NU7441 or irradiation alone (Figure 1g), indicating that the inhibition of DNA-PKcs can increase the efficacy of RT against GBs [67].

**Figure 2:**
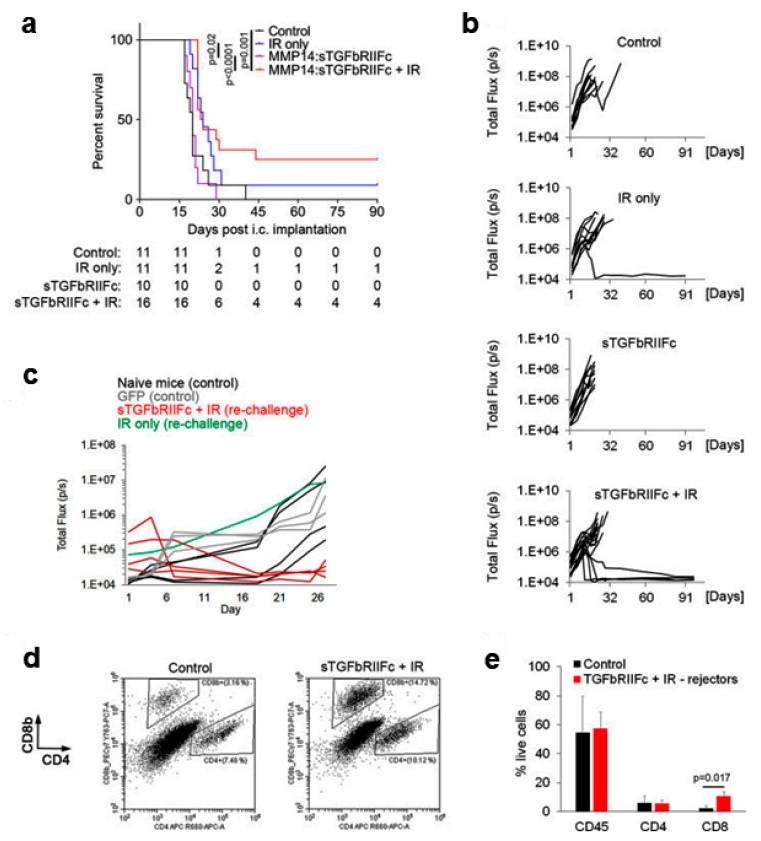
Hematopoietic stem cell (HSC) gene therapy targeting transforming growth factor beta (TGFβ) in combination with IR results in a long-term protection against intracranial GB. (**a**) Survival of mice bearing GL261 intracranial tumors. The table below the graph shows group sizes at the beginning (day 0) and number of animals at risk (e.g., animals that are alive) for indicated days. Pooled data from two independent experiments are shown. Statistical significance was determined by a two-tailed log-rank test. (**b**) Growth curves for individual tumors as obtained by bioluminescence imaging, displaying total flux in photons per second (p/s). (**c**) Mice that rejected GL261 tumors following the first intracranial implantation of cancer cells plus therapy were rechallenged by a second intracranial implantation of GL261 cells at 90 days post-tumor rejection (red and green lines). Mice that have received transplantation of MMP14: GFP-transduced HSCs (gray lines) or naïve mice (black lines) were implanted with GL261 cells intracranially for the first time and used as controls. Tumor growth was quantified by bioluminescence imaging as in (**b**). (**d**,**e**) Representative dot plots showing analysis of CD8+ and CD4+ T cells (**d**) and quantification of immune cells (**e**) in intracranial tumors (control mice as specified in **c**) or brain area corresponding to the cancer cell implantation site (mice that have rejected tumors for the second time in the combination therapy group) by flow cytometry at 3 weeks post-cancer cell implantation. Statistical significance was determined by two-tailed *t*-test (n = 3 and 4, respectively, in first and second experiment for control group; n = 2 per experiment for rechallenged long-term survivors from the combination therapy group) (modified after [68] with permission).

Hematopoietic stem cell (HSC)-derived myeloid cells may home to HGG where they can represent up to 50% of intratumoral cells. Whether hematopoietic stem cells targeting TGFβ may be used to enhance the efficacy of RT has been investigated by Andreou et al. in a preclinical GB model (Figure 2) [68]. Since myeloid cells are ubiquitously present in the body, a lentiviral vector containing the matrix metalloproteinase (MMP) 14 promoter, which is active specifically in tumor-infiltrating myeloid cells as opposed to myeloid cells in other tissues, was used.

Mice were transplanted with HSC transduced with lentiviral vector expressing the soluble fusion protein of the TGFβ receptor II-Fc under regulation of the MMP14 promoter. This TGFβ inhibitory therapy was compared with tumor irradiation, the combination of the two therapies and control. The survival of the mice was monitored to assess the long-term benefits of the therapy. The combination therapy resulted in a significantly longer survival time with respect to the control group and TGFβ blocking monotherapy (Figure 2a). In addition, 25% of mice (four of 16) in the combination therapy group showed long-term survival with complete tumor rejection, compared with only one of 11 mice (9%) in the IR group and 0% in the remaining groups (Figure 2a,b). Ninety days after tumor rejection, the surviving mice were again subjected to intracranial implantation of GL261 cells. While the tumors grew again in the control mice and in the re-implanted mouse belonging to the IR group, the tumors failed to grow in all four of the re-implanted mice belonging to the combination therapy group (Figure 2c). Thus, in contrast to IR monotherapy, combination therapy resulted in long-term protection against tumor re-growth. Analysis of immune cells at the tumor implantation site 3 weeks after intracranial injection of the tumor cells revealed a significantly higher percentage of CD8+ T cells in the four mice in the combination therapy group that had rejected tumors for the second time as compared to the control represented by the mice in which there was tumor re-growth (Figure 2d,e). In contrast, infiltration by total CD45 + hematopoietic cells and CD4+ T cells was similar between the two groups (Figure 2d,e). This suggests that in addition to significantly reducing tumor recurrence and prolonging survival, TGFβ-blocking HSC therapy in combination with IR can provide long-term anticancer protection through the development of memory T cells. In summary, long-term protection from GB could be achieved only after the combined treatment (25% of mice) and was accompanied by a significant increase in CD8+ T cells at the tumor implantation site [68].

**Figure 3:**
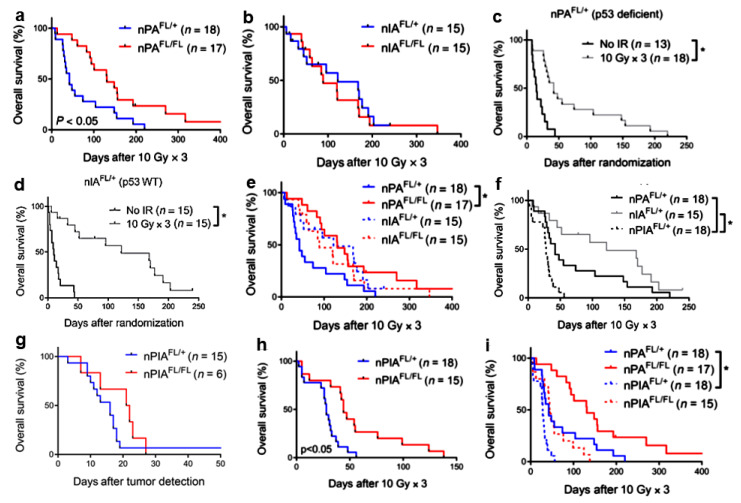
Tumor genotype dictates radiosensitization after Atm deletion in primary brainstem glioma models. (**a**). Deletion of Atm in p53-deficient gliomas improves tumor response to IR. Kaplan–Meier plot of overall survival in tumor-bearing mice after three daily fractions of 10 Gy RT delivered to the whole brain. *, *p* < 0.05 by log rank test. (**b**). Deletion of Atm does not radiosensitize p53 wild-type gliomas. Kaplan–Meier plot of overall survival in tumor-bearing mice after three daily fractions of 10 Gy RT delivered to the whole brain. *, *p* < 0.05 by *t* test (2 tailed). (**c**,**d**). p53 wild-type gliomas with intact ATM function are sensitive to IR. Kaplan–Meier plot of overall survival in (**c**) p53-deficient tumor-bearing nPA^FL/+^ mice or (**d**) p53 wild-type tumor-bearing nIA^FL/+^ mice that received no RT or were treated with three daily fractions of 10 Gy to the whole brain. (**e**). Kaplan–Meier plot comparing the survival of mice with p53 wild-type and p53-deficient gliomas treated with fractionated RT. The animals included in these survival curves are the same animals from the survival studies in panels (**a**) and (**b**). *, *p* < 0.05 by log rank test. (**f**). p53 signaling mediates radiosensitivity of Ink4a/Arf-deficient gliomas. Kaplan–Meier plot comparing the survival of nPA^FL/+^, nIA^FL/+^, and nPIA^FL/+^ mice after treatment with three daily fractions of 10 Gy delivered to the whole brain. Control nPA^FL/+^ and nIA^FL/+^ curves were taken from panels (**a**) and (**b**). *, *p* < 0.05 by log rank test. (**g**–**i**). Deletion of Atm improves the radiation response of gliomas driven by loss of p53 and Ink4a/Arf. (**g**) Kaplan–Meier plot of overall survival in tumor-bearing mice after tumor detection. (**h**) Kaplan–Meier plot of overall survival in tumor-bearing mice after three daily fractions of 10 Gy RT delivered to the whole brain. (**i**) Kaplan–Meier plot comparing the survival of mice with p53-deficient gliomas and gliomas lacking both p53 and Ink4a/Arf that were treated with fractionated RT. Control nPA^FL/+^ and nPA^FL/FL^ curves were taken from panels (**a**,**b**). *, *p* < 0.05 by *t* test (two-tailed) when comparing the mean of two groups or log rank test when comparing survival curves. Tumor genotypes/phenotypes. nPA^FL/+^, nestinTVA p53^FL/FL^ Atm^FL/+^ lacking p53; nPA^FL/FL^, nestinTVA p53^FL/FL^ Atm^FL/FL^ lacking both p53 and ATM; nIA^FL/+^, nestinTVA Ink4a/Arf^FL/FL^ Atm^FL/+/^lacking Ink4a/Arf; nIA^FL/FL^, nestinTVA Ink4a/Arf^FL/FL^ Atm^FL/FL/^lacking both Ink4a/Arf. and Atm; nPIA^FL/+^, nestinTVA p53^FL/FL^ Ink4a/Arf^FL/FL^ Atm^FL/+^ lacking both p53 and Ink4a/Arf (modified after [69] with permission).

The Cre-loxP and replication-competent avian sarcomaleukosis virus long-terminal repeat with splice acceptor–tumor virus A (RCAS-TVA) technology was used to investigate whether the deficiency of the ataxia telangiectasia mutated (ATM) protein in mouse models of DIPG may allow increasing the sensitivity of the tumor to radiation (Figure 3) [69,70]. Primary brain stem gliomas with different genotypes were generated. Nestin-expressing, p53-deficient, ATM competent (nPA^FL/+^), and defective (nPA^FL/FL^) tumors were irradiated with three daily fractions to the brain, each of 10 Gy. The high radiosensitivity of glioma cells in nPA^FL/FL^ mice resulted in a tripling of median survival after RT (Figure 3a, solid red line). These data confirm and extend previous preclinical studies showing that ATM targeting may represent a promising therapeutic approach to improve the HGG response to RT (reviewed in [71]). The deletion of Atm in Ink4a/Arf-deficient, p53 wild-type gliomas (nIA^FL/FL^) did not translate into improved survival after RT in vivo (Figure 3b, solid red line). Thus, the lack of functional ATM radiosensitized primary brain stem gliomas to RT in p53-deficient but not p53 wild-type tumors.

Deland et al. investigated in detail whether the p53 status may affect radio resistance in animal models of brain stem glioma. To that purpose, they compared the radiation response of mutant and wild-type gliomas for p53 in Atm^FL/+^ mice retaining Atm expression. While the survival of mice with p53-deficient tumors tripled after irradiation (median survival increase of 27 days), the survival was augmented 12-fold in mice carrying p53 wild-type tumors (median survival increase of 110 days) (Figure 3c,d (solid gray line)). After three daily fractions of whole brain irradiation, mice with p53 wild-type tumors survived longer than mice with p53 mutant tumors (Figure 3e, dashed blue vs. solid blue). The survival of animals with wild-type gliomas for p53 was comparable to the survival of nPA^FL/FL^ mice with ATM defective tumors (Figure 3e, dashed red vs. solid red). Taken together, the data in Figure 3c–e indicate that wild-type tumors for p53 induced by the Ink4a/Arf mutation exhibit increased sensitivity to RT. These DIPGs are not further radiosensitized by the loss of Atm (Figure 3b). The lack of p53 signaling activity after radiation exposure reduced the radiosensitivity of Ink4a/Arf mutant tumors and caused decreased survival of the affected mice (Figure 3f solid black line).

Unexpectedly, tumors lacking both tumor suppressors were more resistant to IR than those with p53 deficiency alone, as shown by a significant decrease in survival after fractionated RT (Figure 3f, dashed black line). These results indicate that p53 and Ink4a/Arf contribute to tumor cell survival after RT treatment through different mechanisms. Consistent with previous findings, the ATM deletion did not accelerate tumor progression (Figure 3g, solid red line). The deletion of ATM slightly improved the survival of mice whose tumors had been treated with fractionated RT (Figure 3h, solid red line). Taken together, these findings suggest that in primary brain stem gliomas, the p53 status is a critical determinant of tumor response to RT along with inactivation of ATM. Interestingly, the survival of nPIA^FL/FL^ mice undergoing RT (Figure 3i, dashed red line) carrying tumors lacking all three functions p53, Ink4a/Arf, and ATM was comparable to that of nPA^FL/+^ mice (Figure 3i, solid blue line) whose tumors lacked p53 only, indicating that the ATM-defective gliomas induced by loss of p53 and Ink4a/Arf remain relatively radioresistant (Figure 3i). As mentioned above, the deletion of ATM tripled the survival of irradiated mice with p53-deficient tumors and preserved Ink4a/Arf function (Figure 3a, solid red line), while the same genetic modification only increased the survival of 1.5 times in mice carrying tumors lacking both p53 and Ink4a/Arf (Figure 3i, dashed red line). In conclusion, further genetic alterations can contribute to the radiosensitization of tumors with inactivation of ATM and p53. As a consequence, whether pharmacological ATM inhibition can radiosensitize the tumor depends on the status of a number of other, partially identified, genes [69].

**Figure 4:**
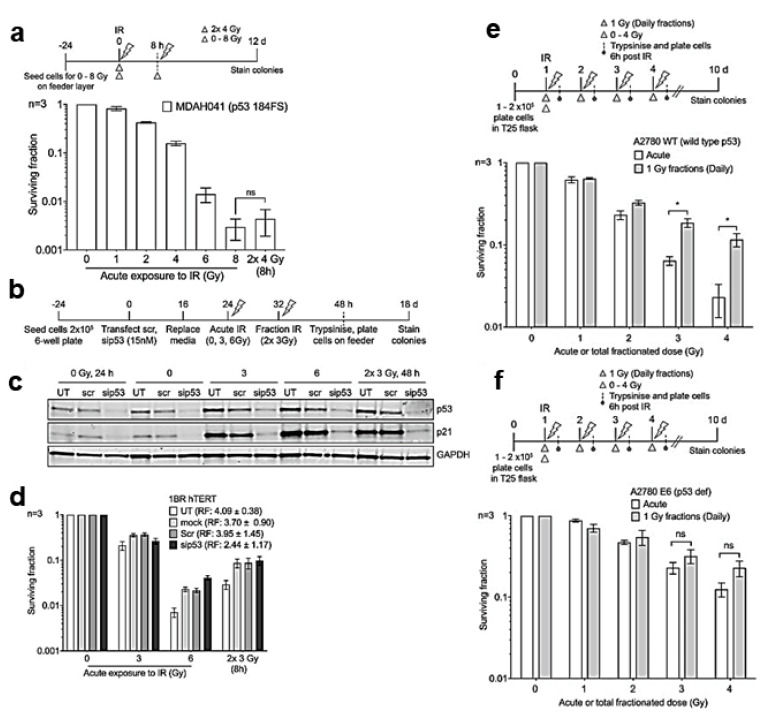
p53 modulates RT fraction size sensitivity in normal and malignant cells. (**a**–**d**). Split-dose recovery is not observed in primary fibroblast with loss of functional p53. (**a**–**d**). Transformed Li-Fraumeni fibroblasts MDAH041 were exposed to either acute or split-dose IR with indicated doses. (**a**) Colony survival assay confirms loss of split-dose recovery (white triangle, single acute dose, and gray triangle represents split dose IR). (**b**) Schema for (**c**) Western blot analysis showing the expression levels of total p53, p21, and loading control GAPDH and (**d**) colony survival of p53 siRNA knockdown in 1BR hTERT cells for the indicated period. UT is untreated, mock represents cells treated with DharmaFECT1 transfection reagent, and Scr is the ON-TARGETplus non-targeting control scramble. RF, the ratio of split-dose to single dose survival, has been compared for each experimental condition. (**e**,**f**). Split-dose recovery is lost in tumor cell lines with mutant p53. Colony survival of tumor cell lines A2780 WT (**e**,**f**) A2780 E6 after exposure to either acute or daily fractionated IR with indicated doses. Top panel in each histogram shows the experimental schema, white triangle represents single acute dose and gray triangle 1 Gy daily fractions. Post IR (6 h) cells were trypsinized and pooled with cells collected from media, plated, and allowed to form colonies. A significant increase in split-dose recovery is observed in p53 WT A2780 (**e**) but not in mut p53 A2780 E6 (**f**) (modified after [72] with permission).

Anbalagan et al. investigated whether the different cell sensitivity to the different sizes of RT fractions may be dependent on the WT p53 status (Figure 4) [72]. Single or fractionated doses of IR were administered to different normal and malignant cell lines, and cell survival was determined by colony counting. A recovery factor (RF) was defined as the ratio of cell survival after fractionated dose to cell survival after single dose and used as a measure of sensitivity to fraction size.

Both normal and tumor cells with WT p53 showed significant recovery after dose fractionation, while Li-Fraumeni fibroblasts with mutant p53 and defective G1/S checkpoint lost the survival improvement due to dose fractionation (Figure 4a). Confirming previous studies that had demonstrated loss of p53 protein expression in MDAH041 cells, Western blot analysis revealed no increase in p21 expression after irradiation, which is indicative of a loss of p53 function. Furthermore, after irradiation, MDAH041 cells were arrested in the G2/M phase, which is consistent with the loss of functionality of the G1/S checkpoint. To further test the hypothesis, p53 knockdown was performed via siRNA in 1BRhTERT cells (Figure 4b). The reduced expression of p53 was confirmed by Western blotting (Figure 4c). The transient reduction of p53 in hTERT1BR cells resulted in a reduction in fractionation recovery observed after 2 × 3 Gy fractions as compared to a single 6 Gy fraction (Figure 4d). Thus, p53 siRNA knockdown in hTERT 1BR fibroblasts reduced cells’ recovery linked to dose fractionation. Ovarian cancer cell isogenic lines (A2780 WT and A2780/E6 where p53 is silenced by HPV E6) were also tested (Figure 4e,f). After fractionated irradiation, p53 WT A2780 WT cells were fraction size sensitive with significant recovery (RF of 6.26 ± 3.54) (Figure 4e). The latter was significantly reduced in p53-defective A2780/E6 cells (RF of 1.82 ± 0.33) (Figure 4f). In agreement with previous data, significant differences in cell cycle phase distributions were observed, with p53 WT A2780 cells mainly in the G1 phase while p53 defective A2780 E6 were mainly in the S/G2 phase. Further experiments confirmed that p53 mutated tumor cells, regardless of NHEJ pathway functionality, are likely insensitive to fraction size [72].

p53 is a factor that also influences the radiosensitization of GICs by ATM inhibitors, which is a phenomenon that, unlike fraction size sensitivity, is less evident when p53 is wild type [73,74].

The effect of astrocyte elevated gene-1 (AEG-1) on the radiosensitivity of glioma cells has been investigated by Zhao et al. [75]. Immunohistochemistry assay found that AEG-1 was generally overexpressed in glioma tissues and was correlated with poor clinicopathological features of glioma patients. AEG-1 knockdown inhibited the proliferation of glioma cells. γ-H2AX foci assay, colony formation assay, and flow cytometry analysis demonstrated that AEG-1 depletion enhanced radiosensitivity and promoted apoptosis as well as cell cycle arrest in the G2 phase of glioma cells treated with IR. The replication factor C5 (RFC5) was identified as a target of AEG-1 by using Affymetrix human gene expression array and RFC5 expression was downregulated in AEG-1 knockdown glioma cells. Mechanistically, AEG-1 knockdown impaired homologous recombination repair activity induced by IR through inhibiting RFC5 expression. Furthermore, the Kaplan–Meier analysis and multivariate Cox regression analysis indicated that high levels of AEG-1 and RFC5 were related to poor prognosis of glioma patients treated with RT [75].

Nile et al. have studied the radiosensitization of glioma cells by inhibitors of glycolysis (2-DG) and mitochondrial function (metformin) [76]. The radiosensitising effects of 2-DG were greatly enhanced by combination with the antidiabetic biguanide, metformin. Metabolomic analysis and cellular bioenergetic profiling revealed this combination to elicit severe disruption of key glycolytic and mitochondrial metabolites, causing significant reductions in ATP generation and enhancing radiosensitivity. Combination treatment induced G2/M arrest that persisted for at least 24 h post-irradiation, promoting apoptotic cell death in a large proportion of cells [76].

The radiosensitizing effect of olanzapine has been evaluated in U87 GB cells [77]. Olanzapine reduced the number of colonies and elevated ROS levels in irradiated cells. The maximum radiosensitizing effect of olanzapine was observed at the concentration of 20 µM [77].

Porphyrins such as 5-aminolevulinic acid-induced protoporphyrin IX selectively accumulate in neoplastic cells and are currently used for the fluorescent-guided surgical resection and photodynamic therapy of HGG. Overall, 5-aminolevulinic acid has been suggested as a radiosensitizer by increasing the oxidative stress in neoplastic cell mitochondria and enhancing the host immune response [78].

A complex consisting of two different nanoparticles, Au-OMV (gold nanoparticles) and OMV (membrane vesicles of E. coli) in combination with RT, produced radiosensitizing and immunomodulatory effects that inhibited the growth of both subcutaneous and orthotopic G261 tumors in C57BL/6 mice [79]. Longer survival was also observed in those mice in comparison to control. A high level of intracellular ROS has been observed in cancer cells exposed to radiation in the presence of the bi-particle complex [79].

The RT minibeam is a type of fractional RT that uses submillimeter beams of radiation [80]. An increase in long-term survival (60%) and a reduction in toxicity compared to conventional (wide beam) irradiation has been reported in glioma-bearing rats treated with minibeam RT [80].

Curcumin has been proposed as a therapeutic agent with radiosensitizing potential in brain tumor therapy [81]. No interaction was found on the survival of U251 human glioma cells after irradiation in combination with curcumin at clinically achievable concentrations. Experimental in vitro and in vivo data together with clinical bioavailability data did not give evidence for a radiosensitizing effect of curcumin. Reported HGG intratumoral curcumin concentrations were too low to either exert their own cytotoxic effect or to synergistically interact with IR. At present, there is neither a biological nor clinical rationale for using curcumin as a radiosensitizer in the therapy of HGG patients [81].

#### 3.2.2. Clinical Studies

Updated guidelines for the diagnosis and management of adult patients with diffuse gliomas have been recently provided by the European Association of Neuro-Oncology (EANO) [61]. The diagnostic component was based on the aforementioned 2016 update of the WHO Classification of Tumors of the Central Nervous System and the subsequent recommendations of the Consortium to Inform Molecular and Practical Approaches to CNS Tumor Taxonomy (Section 2. Diagnosis and prognosis). With regard to therapy, recommendations based on the results from the latest practice-changing clinical trials were formulated, and also guidance for neuropathological and neuroradiological assessment were provided. In these guidelines, the role of the major treatment modalities of surgery, RT, and systemic pharmacotherapy was defined, covering current advances and emphasizing that unnecessary interventions and expenses should be avoided [61].

The hypothesis that the time at which the HGG treatment is carried out may influence the efficacy and/or toxicity of RT has been studied by Sapienza et al. [82]. The time of the day the treatment was performed was classified as morning if ≥50% of the fractions were administered before 12:00 or afternoon if after 12:00. The median follow-up was 10.9 months, and the median OS was 16.5 months. There was no difference in OS between HGG patients treated in the morning or in the afternoon [82].

The recommended phase II dose, safety/tolerability, and preliminary efficacy of combining pembrolizumab, an anti-PD1 monoclonal antibody, with hypofractionated stereotactic irradiation and bevacizumab in patients with recurrent HGGs have been investigated [83]. The combination of hypofractionated stereotactic irradiation with pembrolizumab and bevacizumab in patients with recurrent HGG was generally safe and well tolerated.

Magnetic hyperthermia is a treatment modality where tumor sensitization to RT and CT is explored by heating up tumor cells to 40–45 °C [84]. HGG tumor sensitization by noninvasive inductive magnetic hyperthermia in the treatment of recurrent/progressive patients has been proposed. Magnetic hyperthermia improved OS in primary GB but no significant change in the OS of recurrent GB patients was reported. At present, heterogeneity in the methodology and study design may significantly limit the extent to which conclusions can be drawn on this radiosensitization technique [84].

Hyperbaric oxygen therapy increases the oxygen tension in tissues and, theoretically, it should enhance the efficiency of RT. This subject was recently reviewed by Fernandez et al., who found grade B and C evidence that at pressures of 2 absolute atmospheres, hyperbaric oxygen therapy increased the effectiveness of IR in head and neck tumors and achieved promising results in the local control of HGG [85].

Animal brain-tumor models have demonstrated a synergistic interaction between RT and a ketogenic diet. Metformin has in vitro anti-cancer activity through AMPK activation and mTOR inhibition (Section 3.2.1 Preclinical studies). Porper et al. hypothesized that the metabolic stress induced by a ketogenic diet combined with metformin would enhance IR efficacy. The tolerability and feasibility of this approach was clinically investigated. It was found that the intervention was well tolerated. Higher serum ketone levels were associated with both dietary intake and metformin use. The recommended phase II dose was eight weeks of a ketogenic diet combined with 850 mg metformin twice daily [86].

## 4. The Other Side of the Coin

As yet another confirmation, the expected improved survival has been observed in elderly patients treated with RT in comparison with those receiving the best supportive care alone, with similar survival for patients undergoing conv-FRT (60 Gy/30 fractions) and hypo-FRT (25–40 Gy in 5–15 daily fractions) [87]. Hence, while it has an undeniable role in improving cancer survival, RT has also various negative effects, ranging from mild to severe and including IR-induced meningioma, IR-induced glioma, cavernous malformation, enlarging perivascular spaces, leukoencephalopathy, stroke-like migraine after RT, Moyamoya syndrome, radiation necrosis, IR-induced labyrinthitis, optic neuropathy, retinopathy, and others [88]. Some of them (e.g., cognitive decline) may be influenced by the patient socioeconomic status [89].

The incidence and risk factors associated with IR-induced leukoencephalopathy in long-term survivors of HGG have been investigated by Terziev et al. [90]. A retrospective research for patients with supratentorial HGG treated with focal RT that had a progression-free survival >30 months and available germline DNA was conducted. The median age at the time of RT was 48 years old. The median follow-up after the completion of RT was 79 months. Over half (54.3%) of patients developed leukoencephalopathy, and 35.6% developed consistent symptoms such as subcortical dementia, gait disturbances, and urinary incontinence. The cumulative incidence of leukoencephalopathy was 21% at 12 months, 42% at 36 months, and 48% at 60 months. Age > 60 years, smoking, and the germline SNP rs2120825 at the Peroxisome Proliferator-Activated Nuclear Receptor gamma (PPARg) locus were associated with an increased risk of leukoencephalopathy. PPARg agonists for the prevention and management of late-delayed RT-induced neurotoxicity have been proposed [90].

The incidence and risk factors associated with IR-induced leukoencephalopathy in long-term HGG survivors were studied by Terziev et al. [90]. Patients with supratentorial HGG treated with focal RT, progression-free survival > 30 months, and available germinal DNA were analyzed. The mean age at the time of RT was 48 years. Median follow-up after completion of RT was 79 months. More than half (54.3%) of patients developed leukoencephalopathy, and 35.6% developed symptoms consistent with subcortical dementia, gait disturbance, and urinary incontinence. The cumulative incidence of leukoencephalopathy was 21% at 12 months, 42% at 36 months, and 48% at 60 months. Age > 60 years, smoking, and rs2120825 germline SNP at the PPARg nuclear receptor locus were associated with an increased risk of leukoencephalopathy. PPARg agonists have been proposed for the prevention and management of this late RT-induced complication [90].

Patients with HGG treated with low-activity temporary iodine-125 stereotactic brachytherapy who also had external beam RT were investigated for radiation necrosis side effects [91]. Radiation necrosis was diagnosed using stereotactic biopsy and/or metabolic imaging in 8/75 patients (10.6%). The 1- and 2-year risk of necrosis was 5.1% and 11.7%, respectively, and was mainly determined by the treatment volume rather than by the interval between therapies [91].

Radiosurgery may offer potential for improving the toxicity profile of IR by focused and precise targeting of well-defined tumors under stereotactic immobilization and image guidance [92]. The dosimetric and clinical predictors of IR-induced brain toxicity after single-fraction stereotactic radiosurgery (SRS) or fractionated stereotactic radiosurgery have been surveyed [93]. The risk of radionecrosis after SRS and fractionated SRS was reckoned as a function of dose and volume treated. The use of fractionated SRS appeared to reduce risks of radionecrosis for larger treatment volumes relative to SRS [93]. Published clinical predictors of IR-induced optic nerve/chiasm neuropathy after SRS or fractionated SRS were analyzed, and the optic apparatus normal tissue complication probability and tolerance doses after SRS and fractionated SRS were proposed [94].

Inhibition of neurogenesis due to IR-induced damage in neural stem cells (NSCs) is an important mechanism underlying long-term radiation-induced cognitive impairment [95,96]. After co-culture with GL261 glioma cells, mouse NSCs showed inhibition of proliferation and reduced neurosphere formation and differentiation potential. Irradiated GL261 cells caused greater inhibition and reduction of NSCs than non-irradiated GL261 cells. Furthermore, the addition of exosomes released from GL261 cells to the culture of NSCs inhibited the proliferation of NSCs, suggesting that exosomes derived from cancer cells may be mediators of those intercellular effects. Furthermore, the injection of exosomes released by GL261 cells into the hippocampus of mice caused an inhibition of neurogenesis and cognitive impairment two months later, and exosomes of irradiated GL261 cells induced even greater inhibitory effects.

Recent data have shown that single-fraction irradiation delivered to the whole brain in less than tenths of a second using FLASH radiotherapy (FLASH-RT) does not elicit neurocognitive deficits in mice [97,98]. The capability of FLASH-RT to minimize the induction of radiation-induced brain toxicities has been attributed to the reduction of ROS, raising some concern that this might translate to a possible loss of antitumor efficacy. Montay-Gruel et al. have shown that FLASH and CONV-RT are similarly efficient in delaying GB growth for all tested regimens. Furthermore, only FLASH-RT was found to significantly spare radiation-induced cognitive deficits in learning and memory in tumor-bearing animals after the delivery of large neurotoxic single dose or hypofractionated regimens. Hence, FLASH-RT delivered with hypofractionated regimens may be able to spare the normal brain from radiation-induced toxicities without compromising tumor cure [97,98].

Proton RT has been compared to intensity modulated RT in 90 patients with newly diagnosed HGG by Brown et al. [99]. There was no difference in PFS or OS between study arms. Even though proton RT was not associated with a delay in time to cognitive failure possibly because the aggressive nature of GB overshadows any potentially improved cognitive outcomes by the radiation technique, proton RT was associated with reduced toxicity and patient-reported fatigue [99].

The clinical and therapeutic factors associated with the long-term risk of secondary neoplasm (SN) of the CNS after cranial irradiation in childhood or adolescent cancer survivors have been quantified [100]. After adjustment for reported genetic syndromes and first CNS tumor, the meningioma risk significantly increased with higher IR doses (excess OR per Gy (EOR/Gy) = 1.377; median latency time = 30 years). The risk was higher among youngest individuals at first cancer diagnosis, but it did not vary with follow-up time. On the contrary, IR-related glioma risk (EOR/Gy = 0.049; median latency time = 17 years) decreased over time. There was a significant association between meningioma risk and cumulative doses of alkylating agents but no association with growth hormone therapy [100].

Using a tumor registry, Rodrigues et al. sought to characterize the risk of secondary tumor (SN) development after external beam RT (EBRT) of pediatric low-grade gliomas [101]. A total of 1245 medical records of pediatric patients (aged 0–17 years) from 1973 to 2015 were analyzed. For patients alive 30 years after initial diagnosis of low-grade glioma, the absolute risk of developing SN in the EBRT-treated cohort was 12.61% versus 4.99% in the untreated EBRT cohort. Cumulative incidence curves that were corrected for competing events confirmed higher rates of secondary tumor development in patients treated with EBRT. Thus, it should be considered during treatment planning that RT is associated with an increased risk of future secondary cancers for pediatric survivors of low-grade gliomas [101].

Epilepsy is a common complication in HGG patients after undergoing tumor surgery combined with chemotherapy and/or RT. A model for predicting the risk of epilepsy occurrence in such patients has been provided: a total of 219 patients with gliomas were reviewed. Multivariate analyses revealed that age, WHO glioma classification, CD34, EGFR, O6 methylguanine DNA methyltransferase, and vimentin were predictors of risk of epilepsy occurrence [102]. A nomogram of the risk of epilepsy occurrence was built based on statistically significant variables from the multivariate logistic regression analysis [102].

## 5. Concluding Remarks and Future Perspectives

Due to unacceptable side effects and limited efficacy, escalating RT doses for HGG may be not a viable strategy. To improve the overall effectiveness of currently employed RT toward HGG, several tumor-specific radio-sensitizing drugs are under development [103]. However, radiosensitizing drugs may not easily cross the blood–brain barrier and may possibly exert toxic effects per se when administered systemically. On the other hand, locoregional administration of radiosensitizing drugs (e.g., through convection-enhanced delivery) is of difficult technical implementation and at the moment can be performed in few clinical centers only [104,105]. Inhibiting minimal residual disease progression using low doses of radiation delivered soon after guideline therapies may be an alternative fruitful strategy: in multiple glioma initiating cell-driven animal models that accurately recapitulate the heterogeneity and growth patterns of the patients’ tumors, it was recently demonstrated that ultra-hyper-FRT started at early stages of tumor progression resulted in a significant delay in tumor progression and an improvement in animal survival [55]. Those preclinical results challenge the dogma that few ablative doses are more effective than many small doses for the RT of HGG and provide the rationale for investigating the currently employed treatment regimen extended with an ultra-hyper-FRT schedule for HGG patients.

## Abbreviations

AEG-1, astrocyte elevated gene-1; ALT, alternative lengthening of telomeres; ATM, ataxia telangiectasia mutated; AuNPs, gold nanoparticles; CNS, central nervous system; CT, computed tomography; DAG-G, diffuse astrocytic glioma, IDH-wildtype, with molecular features of glioblastoma; DIPG, diffuse intrinsic pontine glioma; DNA-PK, DNA-dependent protein kinase; EANO, European Association of Neuro-Oncology; EBRT, external beam RT; ECOG, Eastern Cooperative Oncology Group; EGFR, epidermal growth factor receptor; EGFRvIII, epidermal growth factor receptor variant III; FDA, Food and Drug Administration; GB, glioblastoma; GIC, glioma initiating cell; GTV, gross tumor volume; HGG, high-grade glioma; HSC, hematopoietic stem cell; IDH, isocitrate dehydrogenase; IR, ionizing radiation; KPS, Karnofsky performance status; MMP, matrix metalloproteinase; MRI, magnetic resonance imaging; NLRP6, NLR family pyrin domain containing 6; NSC, neural stem cell; OMVs, outer-membrane vesicles; OS, overall survival; PFS, progression-free survival; PPARg, peroxisome proliferator-activated nuclear receptor gamma; PS, performance status; PTV, planned target volume; RF, recovery factor; RFC5, replication factor C5; ROS, reactive oxygen species; RT, radiotherapy; SN, secondary neoplasm; SP1, specificity protein 1; SRS, stereotactic radiosurgery; TERT, telomerase reverse transcriptase; TGF, transforming growth factor; TMZ, temozolomide; TTFs, tumor treating fields; TUNEL, terminal deoxynucleotidyl transferase-mediated deoxyuridine triphosphate nick end labeling; WHO, World Health Organization.
